# Recurrent Aphthous Stomatitis Improved after Eradication Therapy for *Helicobacter pylori*

**DOI:** 10.1155/2021/5543838

**Published:** 2021-03-30

**Authors:** Yinglin Gao, Nikhil Gupta, Maisa Abdalla

**Affiliations:** ^1^Internal Medicine, Loma Linda University Medical Center, Loma Linda, CA, USA; ^2^Gastroenterology Department, Loma Linda University Medical Center, Loma Linda, CA, USA

## Abstract

*Helicobacterpylori* (*H*. *pylori*) is a Gram-negative bacterium that colonizes gastric mucosa and is often transmitted through direct contact with saliva, contaminated food or water, and vomit. The majority of the infected individuals remain asymptomatic for a long period. Infection with *H. Pylori* often presents with dyspepsia, nausea, frequent belching, bloating, abdominal discomfort, burning abdominal pain, and peptic ulcer. A potential association between *H*. *Pylori* and recurrent aphthous stomatitis was previously reported; however, the presence of causative relationship between the two remained controversial. We are presenting a case of recurrent aphthous stomatitis of twenty-four-year history resolved after *H*. *pylori* treatment.

## 1. Introduction

Recurrent aphthous stomatitis (RAS) is a recurrent painful ulcerative disorder that commonly affects the oral mucosa. The diagnosis of RAS is clinical, and the etiology remains unclear. Some predisposing factors include trauma, hormonal changes, diet, nutritional deficiency, celiac disease, and immunological disorders. Nutritional deficiency that could potentially lead to RAS includes decreased level of iron, vitamins B3 and B12, vitamin C, and folic acid [1].

*Helicobacter pylori* (*H*. *pylori*) is a Gram-negative bacterium that colonizes gastric mucosa and is often transmitted through direct contact with saliva, contaminated food or water, and vomit. The majority of infected individuals remain asymptomatic for a long period. Consequently, it can lead to various gastrointestinal disorders such as gastritis, peptic ulcers, and even malignancy. In fact, *H* pylori leads to roughly 89% of all gastric cancers [2]; therefore, early recognition and eradication is of great importance. Although *H*. *pylori* infection has been suggested to be involved in the pathogenesis of RAS, this association is debatable. We present a case in which RAS resolved with *H. pylori* eradication therapy suggesting possible association between RAS and *H. pylori.*

## 2. Case Presentation

**Figure 1:**
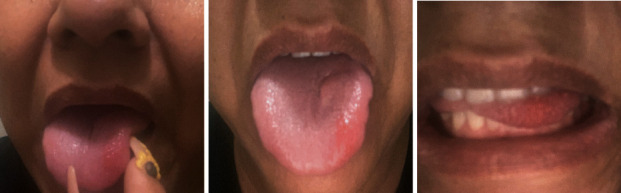
Aphthous stomatitis noted over the left anterolateral tongue (pictures were provided by the patient).

Our patient is a 49-year-old Hispanic immigrant female with a 24-year history of painful RAS in the oral cavity (Figure 1). Her symptoms were associated with nausea and odynophagia. She reports that the sores last approximately one week at a time and can present with as many as thirteen at a single time. There was never a period of time when she had not had an oral ulcer present for the past 20 years and often she would have an average of 5–6 oral ulcers at any given time. Within the past 20 years, she has seen many doctors before immigrating to the US, but it is unclear what kind of treatment she tried in the past and she never got a formal diagnosis of her problem as well. She has only been controlled with pain medication for symptom relief. She also reports a longstanding history of intermittent postprandial bloating and a history of constipation. She denies diarrhea, hematochezia, and personal or family history of colon cancer or gastric cancer. Other past medical history includes anxiety, history of stroke, and hypertension. Her anxiety is very well controlled. She denies drinking or smoking. Differentials such as lupus, Crohn's, ulcerative colitis, Behcet syndrome, HIV, deep mycosis, tuberculosis, syphilis, celiac disease, vasculitis, drug-induced injury (NSAIDs or alendronate), and genital ulcers were all considered. The patient had an extensive lab workup for her aphthous ulcers by her PCP (Table 1) including antinuclear antibody, tissue transglutaminase IgA, HIV 1&2 Ab, and HSV-1 IgM, which were all negative. HSV-1 IgG was positive, but the patient had received multiple courses of acyclovir without resolution of her symptoms. The nutrition panel revealed vitamin B and zinc deficiencies for which a three-month supplementation was given without improvement in her symptoms. Repeat vitamin levels were ordered, but the patient did not follow up. She was seen by a dermatologist who recommended triamcinolone paste to affected areas in her mouth without help. She was then referred to gastroenterology for further workup.

**Figure 2:**
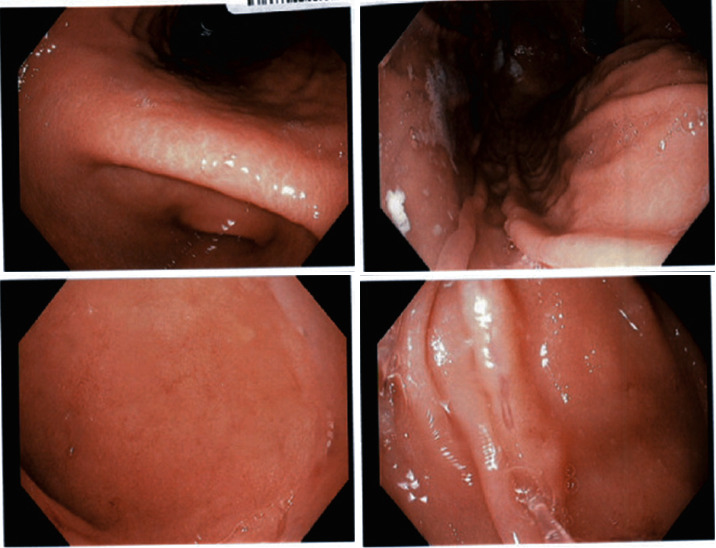
Endoscopic appearance of stomach.

For her physical exam, oral cavity revealed normal lips, normal dentition, and occlusion; two aphthous ulcers on the right buccal mucosa, approximately 0.5 cm in size were seen. No drainage or purulent discharge was noted so the ulcer was not swabbed for examination. Other reviews of systems including skin, eye, lymphatics, cardiovascular, abdomen, thyroid, neurologic, and respiratory systems were all normal. Esophagogastroduodenoscopy was done and revealed erythematous gastric mucosa (Figure 2). The biopsy of this area was positive for *H. Pylori* infection and mild active chronic gastritis. She was then prescribed quadruple therapy consisting of a proton pump inhibitor, tetracycline, metronidazole, and bismuth subsalicylate. After completion of the therapy, she reported simultaneous resolution of RAS. *H. pylori* stool antigen was performed after completion of quadruple therapy and confirmed eradication. She was seen in follow-up five months later and denies the presence of RAS which has not happened for the last 20 years.

## 3. Discussion

It was estimated that the prevalence of *H. Pylori* infection is approximately 20 percent for people less than 30 years and 50 percent for those greater than 60 years in the United States [3]. The exact etiologies for RAS is unclear and could be multifactorial; risk factors could be categorized into local, systemic, immunologic, positive family history with genetic predisposition, allergic, nutritional problems such as B12 deficiency, microbial factors, and anxiety or immunosuppressive drugs [4, 5]. A potential association between *H. Pylori* and recurrent aphthous stomatitis was previously reported; however, the presence of causative relationship between the two remained controversial. It was previously hypothesized *H. pylori* could modify the immune response directly and release proinflammatory mediators, leading to the irritation of surface epithelial cells. [6] In addition, *H. pylori* could potentially multiply in macrophages, dendritic cells, and epithelial cells and then indirectly lead to vitamin B deficiency, which would then cause changes in the epithelium of the tongue and buccal mucosa that would lead to mucosal bleeding and glossitis, given its important role in DNA synthesis [6].

As mentioned previously, the association between *H. pylori* and RAS is unclear. Multiple studies in the past have shown conflicting results regarding the association between RAS and *H. pylori*. In our case, the inability to identify other causes for the patient's 20+ year history of recurrent aphthous ulcers, the lack of response to other commonly used therapies (acyclovir, topical corticosteroids, and vitamin supplementations), and the rapid and sustained resolution of all oral ulcers after completing *H. pylori* treatment support the degree of clinical correlation between recurrent aphthous stomatitis and *H. pylori.* In a prospective study by Karaca et al., a total of 23 patients with RAS were assessed for the association with *H. pylori* and the effect of eradication therapy on the recurrence. All patients underwent endoscopy and had gastric biopsies evaluated for *H. pylori*. The ones with confirmed *H. pylori* were followed up for up to a year after treatment. The result revealed a statistically significant decrease in recurrence rate of RAS with eradication therapy [7]. Similarly, Tas et al. also evaluated 46 patients with aphthous lesions, and they also underwent endoscopic biopsy to evaluate *H pylori*. It was found *H. pylori* positive in 30 patients and negative in 16 patients. The 30 patients with positive results received treatment and were followed up. The result revealed that vitamin B12 levels were significantly increased in the *H. pylori*-eradicated group (*P*=0.001); however, no significant change was found in the control group (*P*=0.638). In addition, the average number of aphthous lesions (per 6 months) of *H. pylori*-eradicated group was significantly decreased after eradication (*P*=0.0001) as compared to the control group (*P*=0.677) [8]. Furthermore, in a meta-analysis study in 2014, Li et al. assessed seven case-control studies containing 339 cases and 271 controls. The result revealed that patients with RAS had significantly higher rates of *H. pylori* infection than non-RAS controls. They concluded that *H. Pylor*i is associated with an increased risk of RAS [9].

On the contrary, few studies were found to have no association between *H pylori* infection and RAS. In a study by Mansour-Ghanaei et al., toothbrush was used to obtain the oral aphthous ulcer from 50 patients that aged from 18 to 60 years old, and samples were sent for both DNA PCR and ELISA tests to evaluate for *H. Pylori*. The result revealed 26 patients (52%) had positive ELISA tests, and only one patient (2%) was positive for DNR PCR [10]. Similarly, Fritscher et al. also evaluated 105 children and adolescents, where 53 patients were found to have RAS and 52 without it. They analyzed samples obtained by swabbing aphthous lesions, oral mucosa, and dental plaque. These samples were then sent for nested PCR to be evaluated for *H. pylori*, and results were similar to the study conducted by Fritscher et al. [11]. Moreover, Iamaroon et al. evaluated the association of *H. pylori* and RAS by nested PCR, the oral samples were also obtained by using toothpicks to brush the ulcer surfaces and/or the dorsum of the tongue in 22 patients with RAS that ages ranging from 12–36 years. The results revealed *H. pylori* does not play a role in the pathogenesis of RAS [12]. The negative associations found in the above studies could be due to the level of bacteria in the oral cavity under undetectable level by one round of PCR (Kignel et al. 2005), the location in the mouth for collection, or sensitivity of PCR difference with saliva vs gastric biopsies [13]. Kignel pointed out that the location mouth used for the collection of the samples can influence the prevalence of the microorganism [13]. This statement was further supported by Song et al. (2000), who found 82% prevalence in the molar region, 64% in premolar region, and 59% in the incisor region [14]. Besides the location, the PCR sensitivity difference in gastric biopsy sample and saliva sample remained unclear, and the evidence is limited. Sekhar et al. found sensitivity and specificity of PCR for saliva were found to be 80% and 70% [15] which is lower compared to gastric biopsy in detecting *H. pylori* which might explain the potential variance in result [16].

## 4. Conclusion

The debate on the association between RAS and *H. pylori* remains. The variable results could stem from the broad inclusion and exclusion criteria each study had, small sample sizes, demographics, and the various detection methods that differ among studies. This association requires further investigation. Despite the ongoing debate between association between *H. pylori* and RAS, *H. pylori* should still always be considered as one of the potential factors contributing to recurrent aphthous stomatitis. Eradication of *H. Pylori* could potentially make a drastic impact on a patient's quality of life and help prevent potential detrimental consequences of untreated *H. pylori* infection such as lymphoma or other gastric malignancies.

## Figures and Tables

**Table 1:** Lab work in diagnosis of the etiology of the recurrent aphthous stomatitis.

CBC	WBC	9.5 th/*μ*L	3.8–10.8 th/*μ*L
	Hgb	13.5 g/dL	11.7–15.5 g/dL
	Hct	42.1%	35–45.0%
	MCV	86.1 fL	80–100 fL
	Platelet	301 th/uL	7.5–12.5 th/uL
	Absolute lymphocyte	3891 cells/uL	200–950 cells/uL

CMP	Na	143 MEQ/L	135–146 MEQ/L
	K	5.0 MEQ/L	3.5–5.3 MEQ/L
	Cl	106 MEQ/L	98–110 MEQ/L
	CO2	32 MEQ/L	20–32 MEQ/L
	Albumin	4.4 g/dL	3.6–5.1 g/dL
	Bilirubin	0.3 g/dL	0.2–1.2 g/dL
	AST	27 IU/L	10–35 IU/L
	ALT	37 IU/L	6-29 IU/L
	BUN	17 mg/dL	7–25 mg/dL
	Creatinine	0.83 mg/dL	0.5–1.10 mg/dL
	Calcium	12.1 mg/dL	8.6–10.2 mg/dL

Micronutrients/vitamins	B12	484 pg/mL	254–1320 pg/mL
	B1	<7 nmol/L	8–30 nmol/L
	B6	<2 ng/*μ*L	2.1–21.7 ng/*μ*L
	B2	43.5 nmol/L	6.2–39 nmol/L
	B3	<20 ng/mL	<20–30000 ng/mL
	Vitamin D 25 (OH)	19 ng/mL	30–100 ng/mL
	Transthyretin	27.7 mg/dL	20–40 mg/dL
	Folate	13.1 ng/mL	3.1–17.5 ng/mL
	TIBC	370 mcg/dL	250–450 mcg/dL
	Ferritin	147.8 ng/mL	8–252.0 ng/mL
	Iron	48 ng/mL	50–170 ng/mL
	Zinc	53 mcg/dL	60–130 mcg/dL

Serologies/autoimmune work up	ANCA	Negative	Negative
	ANA	Negative	Negative
	Rheumatoid factor	Negative	Negative
	TTG-IGA	Negative	Negative
	Lupus panel	Negative	Negative
	SSA/SSB	Negative	Negative
	T4	1.02 ng/dL	0.76–1.46 ng/dLf
	TSH	1.12 mIU/L	0.358–3.740 mIU/L
	A1c	6.2%	<5.5%

Infectious work up	HIV	Negative	Negative
	HSV 1	Positive abs	Negative
	Hepatitis C ab	Negative	Negative
	H pylori (stool antigen)	Negative (after eradication)	Negative

Lipid Panel	Total cholesterol	217 mg/dL	<200 mg/dL
	HDL	67 mg/dL	>50 mg/dL
	Triglyceride	263 mg/dL	<150 mg/dL

CBC: complete blood count, WBC: white blood cell, Hgb: hemoglobin, Hct: hematocrit, MCV: mean corpuscular volume. CMP: comprehensive metabolic panel. AST: aspartate aminotransferase. ALT: alanine transaminase. TIBC: total iron-binding capacity. ANCA: antineutrophil cytoplasmic antibodies. ANA: antinuclear antibodies. TTG-IGA: tissue transglutaminase-immunoglobulin A. SSA/SSB: Sjögren's-syndrome-related antigen A/Sjögren's-syndrome-related antigen B. TSH: thyroid stimulating hormone. HIV: human immunodeficiency virus.

## Data Availability

The data used to support this study are available within the article.
